# Psyllium Overdose During Bowel Preparation Leading to Pneumatosis Intestinalis

**DOI:** 10.14309/crj.0000000000001820

**Published:** 2025-09-10

**Authors:** Parker Penny, Steven Lorch

**Affiliations:** 1 Morsani College of Medicine, University of South Florida, Tampa, FL

**Keywords:** colonoscopy, psyllium, SBO, pneumatosis, bowel preparation

## Abstract

Bowel preparation for colonoscopy can lead to severe complications if mismanaged. We present a case of a 53-year-old man who developed small bowel obstruction and pneumatosis intestinalis due to a medication error, confusing polyethylene glycol with psyllium. While generally safe, psyllium is known to cause obstruction if ingested in large quantities. While the patient responded well to conservative treatment, this case demonstrates a serious complication of laxative misuse. Clear patient education is essential for the safety and efficacy of bowel preparation before colonoscopy.

## INTRODUCTION

Bowel preparation (prep) is essential for successful colonoscopy yet is a substantial burden for patients.^1^ Although the lack of tolerability and palatability of prep agents is most common, there are more serious complications of bowel prep that have been described. One study showed the rate of colonoscopy cancellation due to prep issues to be approximately 2% with the most common complications related to gastrointestinal intolerance and fluid/electrolyte imbalances resulting in dizziness or falls.^2^ Successful bowel prep is achieved through adequate patient education with clear instructions for laxative use.^3,4^ In this study, we present a case of medication error during bowel preparation that led to a small bowel obstruction (SBO) with pneumatosis intestinalis.

## CASE REPORT

A 53-year-old man with a medical history of well-controlled hypertension presented to the emergency department with abdominal pain that began overnight. He had severe nausea, cramping, and abdominal distention with a small amount of nonbilious, nonbloody vomiting and without diarrhea or fever. He had been prescribed a standard regimen of polyethylene glycol (PEG) powder to take the day before in preparation for his screening colonoscopy. However, the patient was confused on the instructions and accidently replaced PEG with psyllium (of which both medications have similar brand names). He took his first dose of 2 tablespoons of psyllium dissolved in 8 oz of water at 7 pm the night before. He repeated this dosing every hour until 11 pm when he developed abdominal pain, distension, and nausea. At this point, he had consumed 10 tablespoons of psyllium, approximately 34 g of psyllium. He was having bowel movements, but they were formed. He continued the hourly dosing for 2 more hours with increasingly severe symptoms. He continued to have bowel movements throughout the night. In total, he consumed approximately 14 tablespoons (47.6 g) of psyllium over a 6-hour period. He arrived at the emergency department at 9 am

**Figure 1.**
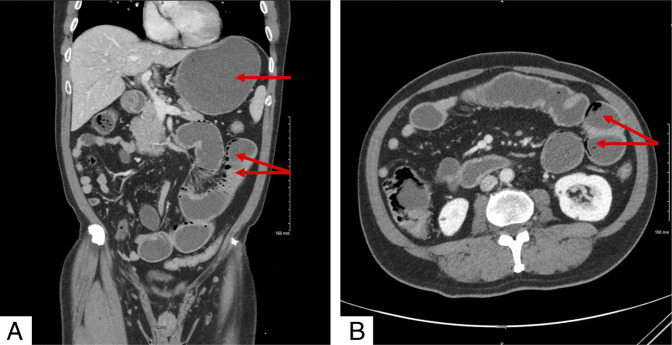
Computed tomographyof the abdomen and pelvis coronal (A) and axial (B) views showing pneumatosis intestinalis and marked distension of the stomach. The red arrows indicate the areas of interest.

**Figure 2.**
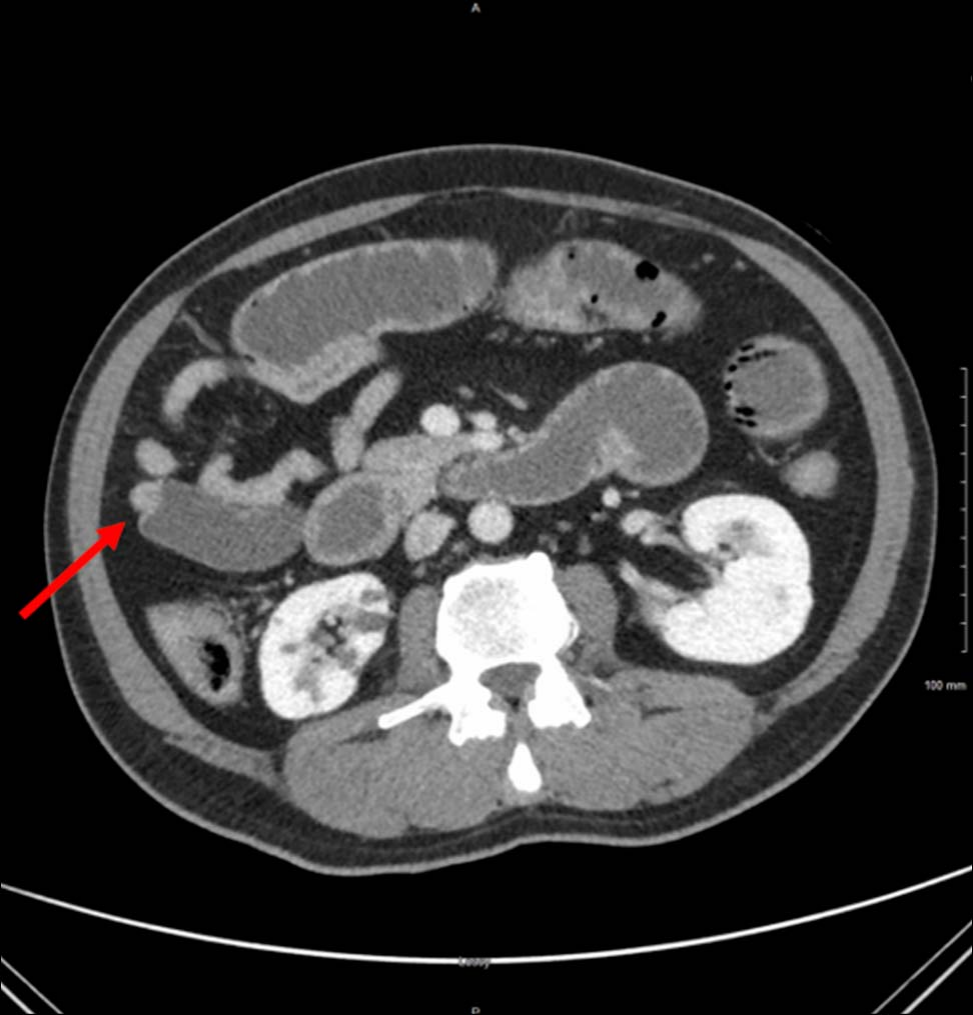
Small bowel transition point in the right mid-abdomen. The red arrow indicates the area of interest.

He was hemodynamically stable on arrival. Physical examination showed a moderately distended tender abdomen without guarding or rebound pain. Significant laboratory results are presented in Table 1. A computed tomography scan of the abdomen and pelvis with intravenous contrast was performed revealing an acute SBO with associated pneumatosis intestinalis (Figure 1). There was also marked distension of the stomach with liquid food material. A transition point was visualized in the right midabdomen (Figure 2).

**Table 1.** Significant laboratory values at initial presentation

Test	Result	Reference range
White blood cell	12.24	4.60–10.20 10*3/µL
Creatinine	1.10	0.72–1.25 mg/dL
Blood urea nitrogen	15	8.4–25.7 mg/dL
Aspartate aminotransferase	34.0	5.0–34.0 U/L
Alanine aminotransferase	47	5–55 U/L
Anion gap	18	5–13 meq/L
Lactate	3.6	0.5–2.2 mmol/L

The patient was given a 1 L bolus of 0.9% NaCl and put on maintenance fluids. He was also given acetaminophen, diphenhydramine, morphine, and ondansetron, and IV pantoprazole. Acute care surgery was consulted and a nasogastric (NG) tube was placed, confirmed with X-ray, and set to intermittent wall suction. 1,400 CC of gelatinous output was initially removed from the stomach with subsequent resolution of the patient's nausea and pain. Lactate continued to normalize throughout the day to a level of 1.4 mmol/L, and an additional 300 CC of NG output was recorded.

The next day, a 500 mL PEG suspension was instilled into the stomach through NG tube for 2 h. This was repeated once for a total of 2 doses. The patient's symptoms and abdominal distension were markedly improved by hospital day 3, and the NG tube was removed. The patient was advanced to a regular diet and discharged on hospital day 4.

## DISCUSSION

Colonoscopy is a safe diagnostic and therapeutic modality for colorectal disease. However, millions of colonoscopies are performed annually in the United States alone and the sheer number of procedures leads to inevitable complications.^5^ Most of the research regarding these complications is focused on intraoperative and postoperative adverse events with sparse literature on the risks associated with preoperative bowel preparation.^6–8^

This case demonstrates a significant complication of bowel preparation stemming from a medication error. The patient presented with a SBO and pneumatosis intestinalis, which is an indicator of advanced bowel wall ischemia.^9^ If the patient would have waited longer to seek care, the ischemia may have progressed to the point of requiring a bowel resection. However, while pneumatosis is a concerning radiographic sign, it does not always indicate complete transmural ischemia and can be reversible if treated promptly. This was the case in our patient, whose symptoms resolved with conservative treatment.^10^

Our patient misinterpreted the bowel preparation instructions given to him and confused 2 common household laxatives: PEG and psyllium. Psyllium is widely used fiber supplement and typically is very safe. However, while rare, psyllium causing intestinal obstruction is a well-reported complication of the bulk-forming laxative. There are reports of small bowel, large bowel, esophageal, and even gastric pouch obstructions.^11–13^ It is also not always acute psyllium ingestion as in our case; chronic supplementation has also been shown to lead to obstruction.^14,15^ When mixed with water, the psyllium forms a nondigestible hydrogel. If the patient does not have adequate fluid intake, however, it can become a bezoar leading to intestinal distension and subsequent obstruction.^16,17^

This is not the first report of medication error during bowel preparation leading to an adverse patient outcome. In 2017, the Institute for Safe Medication Practices Canada released a safety bulletin detailing a patient case requiring hemodialysis where a pharmacy filled a 2 L prescription of propylene glycol instead of the ordered PEG suspension.^18^ Our case highlights the importance of clear patient education for bowel preparation in order to prevent medication errors. Ensuring patients understand the correct use of prescribed laxatives enhances patient safety.

## DISCLOSURES

Author contributions: P. Penny had substantial contributions to the conception or design of the work and drafting the work. S. Lorch reviewed it critically for important intellectual content and gave final approval of the version to be published and is in agreement to be accountable for all aspects of the work as its guarantor in ensuring that questions related to the accuracy or integrity of any part of the work are appropriately investigated and resolved.

Financial disclosure: None to report.

Informed consent was obtained for this case report.

## ABBREVIATIONS:

NG: nasogastric
PEG: polyethylene glycol
Prep: preparation
SBO: small bowel obstruction
